# Long-term effects of immunotherapy with a brain penetrating Aβ antibody in a mouse model of Alzheimer’s disease

**DOI:** 10.1186/s13195-023-01236-3

**Published:** 2023-05-02

**Authors:** Tobias Gustavsson, Nicole G. Metzendorf, Elin Wik, Sahar Roshanbin, Ulrika Julku, Aikaterini Chourlia, Per Nilsson, Ken G. Andersson, Hanna Laudon, Greta Hultqvist, Stina Syvänen, Dag Sehlin

**Affiliations:** 1 Department of Public Health and Caring Sciences, Uppsala University, Uppsala, Sweden; 2 Department of Pharmacy, Uppsala University, Uppsala, Sweden; 3 Department of Neurobiology, Care Sciences and Society, Division of Neurogeriatrics, Karolinska Institutet, Stockholm, Sweden; 4 BioArctic AB, Stockholm, Sweden

**Keywords:** Alzheimer’s disease (AD), Immunotherapy, Amyloid-β (Aβ), Monoclonal antibody, Blood–brain barrier (BBB), Transferrin receptor (TfR)-mediated transcytosis

## Abstract

**Background:**

Brain-directed immunotherapy is a promising strategy to target amyloid-β (Aβ) deposits in Alzheimer’s disease (AD). In the present study, we compared the therapeutic efficacy of the Aβ protofibril targeting antibody RmAb158 with its bispecific variant RmAb158-scFv8D3, which enters the brain by transferrin receptor-mediated transcytosis.

**Methods:**

*App*^*NL−G−F*^ knock-in mice received RmAb158, RmAb158-scFv8D3, or PBS in three treatment regimens. First, to assess the acute therapeutic effect, a single antibody dose was given to 5 months old *App*^*NL−G−F*^ mice, with evaluation after 3 days. Second, to assess the antibodies’ ability to halt the progression of Aβ pathology, 3 months old *App*^*NL−G−F*^ mice received three doses during a week, with evaluation after 2 months. Reduction of RmAb158-scFv8D3 immunogenicity was explored by introducing mutations in the antibody or by depletion of CD4^+^ T cells. Third, to study the effects of chronic treatment, 7-month-old *App*^*NL−G−F*^ mice were CD4^+^ T cell depleted and treated with weekly antibody injections for 8 weeks, including a final diagnostic dose of [^125^I]RmAb158-scFv8D3, to determine its brain uptake ex vivo. Soluble Aβ aggregates and total Aβ42 were quantified with ELISA and immunostaining.

**Results:**

Neither RmAb158-scFv8D3 nor RmAb158 reduced soluble Aβ protofibrils or insoluble Aβ1-42 after a single injection treatment. After three successive injections, Aβ1-42 was reduced in mice treated with RmAb158, with a similar trend in RmAb158-scFv8D3-treated mice. Bispecific antibody immunogenicity was somewhat reduced by directed mutations, but CD4^+^ T cell depletion was used for long-term therapy. CD4^+^ T cell-depleted mice, chronically treated with RmAb158-scFv8D3, showed a dose-dependent increase in blood concentration of the diagnostic [^125^I]RmAb158-scFv8D3, while concentration was low in plasma and brain. Chronic treatment did not affect soluble Aβ aggregates, but a reduction in total Aβ42 was seen in the cortex of mice treated with both antibodies.

**Conclusions:**

Both RmAb158 and its bispecific variant RmAb158-scFv8D3 achieved positive effects of long-term treatment. Despite its ability to efficiently enter the brain, the benefit of using the bispecific antibody in chronic treatment was limited by its reduced plasma exposure, which may be a result of interactions with TfR or the immune system. Future research will focus in new antibody formats to further improve Aβ immunotherapy.

**Supplementary Information:**

The online version contains supplementary material available at 10.1186/s13195-023-01236-3.

## Background

Already decades before onset of clinical symptoms of Alzheimer’s disease (AD), an imbalance between production and clearance of the amyloid-β (Aβ) protein leads to aggregation of the protein into soluble oligomers and protofibrils, eventually forming insoluble fibrils, which precipitate into the brain tissue as more or less densely packed plaques. Many late clinical trials of both secretase inhibitors and immunotherapy have failed, but two antibodies, *aducanumab* (Aduhelm) [1] and *lecanemab* (Leqembi) [2], have been conditionally approved by the American Food and Drug agency (FDA) for treatment of patients with early AD. *Lecanemab*, which was developed in our lab [3], was recently reported to achieve a reduction in both amyloid burden and the rate of cognitive decline in large clinical AD trials [4–6]. Two additional antibodies, *gantenerumab* and *donanemab*, are currently evaluated in phase 3 clinical trials. Common for all these four antibodies is the choice of Aβ as target. While earlier generations of immunotherapies were based on antibodies with affinity for monomeric Aβ, which is abundantly present in both blood and brain, these four antibodies bind preferentially to aggregated or truncated, pathological forms of the Aβ protein [1, 3, 7–10].

A major drawback for all of the therapeutic antibody candidates mentioned above is that antibodies in general do not readily cross the blood–brain barrier (BBB), and therefore achieve limited brain concentrations. Receptor-mediated transcytosis (RMT) is an emerging strategy to increase brain delivery of therapeutic antibodies, to improve their therapeutic effect, and to reduce side effects related to high peripheral drug concentrations. The transferrin receptor 1 (TfR1) has been widely used for this purpose [11] and a fusion of *gantenerumab* and a TfR1 binding antibody fragment-based “brain shuttle” recently entered clinical trials [12]. Various designs of TfR1-targeted bispecific antibody constructs have been explored and medium affinity, with monovalent TfR1 interactions, have been reported to favor efficient transport into the brain [13, 14]. Moreover, the location of the TfR1 binding moiety in relation to the antibody’s Fc domain appears to be important to avoid blood cell interactions leading to adverse first infusion reactions [15].

We have designed a bispecific antibody based on the Aβ protofibril selective mAb158 [3] (murine parent of *lecanemab*), recombinantly fused with a single chain variable fragment (scFv) of the TfR1 antibody 8D3 fused to each of the light chains. Despite its bivalent design, RmAb158-scFv8D3 has a preference for monovalent binding to TfR1, which results in highly efficient BBB transport [16–18]. Besides increasing total brain drug concentration, the TfR1-mediated entry through the capillaries of the whole volume of the brain results in a much wider brain distribution of the bispecific antibody compared with the unmodified RmAb158 [19, 20]. Consequently, a single dose of RmAb158-scFv8D3 was ten-fold more efficient than RmAb158 in clearing soluble Aβ aggregates from the brain of AD transgenic mice (tg-ArcSwe) [20–22]. Although this mouse model develops Aβ plaques with a structure similar to those found in the AD brain, transgenic expression of APP has been associated with various types of artifacts that can affect the physiology of the mouse [23]. Instead, there is an increased interest in using new mouse models such as *App*^*NL−G−F*^*,* a knock-in mouse model where mouse APP is substituted with a humanized Aβ sequence and the Swedish, Arctic, and Beyreuter/Iberian APP mutations [24].

Compared with a small bispecific antibody construct with only one scFv8D3, the format of RmAb158-scFv8D3 seems to favor longer residence in the capillary walls, which could be a sign of partial bivalent TfR1 interactions in the capillary endothelium, potentially leading to less efficient BBB transport [25]. How this affects brain uptake after repeated dosing remains to be studied. Another potential issue with repeated injections of bispecific antibody constructs is the risk of immunological reactions caused by linkers or domains originating from foreign species. Previous preclinical studies using bispecific antibodies in long-term studies have either suppressed the immune response by CD4^+^ T cell depletion prior to administration [15] or registered an immune reaction in the form of anti-drug antibodies (ADA) [26]. ADA is a response to foreign substances, either in the form of an amino acid sequence from a different species or a foreign structural element, which can be formed e.g. due to protein aggregation.

Here, we have explored RmAb158-scFv8D3 in various different therapeutic regimens in the *App*^*NL−G−F*^ model of Aβ pathology. In addition to studying the therapeutic effects of the antibody, we have evaluated the consequences of repeated injections with and without suppression of the immune system.

## Methods

### Animals

Wild type (wt) and *App*^*NL−G−F*^ mice, expressing mouse amyloid precursor protein (*APP*) with a humanized Aβ sequence and with the Swedish (KM670/671NL), Arctic (E693G), and Iberian (I716F) APP mutations [24], were used in the study. All experimental procedures were approved by the Uppsala County Animal Ethics board (5.8.18–13,350/2017 and 5.8.18–20,401/2020), carried out according to regulations of the Swedish Animal Welfare Agency, and complied with the European Communities Council Directive of 22 September 2010 (2010/63/E.U.).

### Antibodies — design, production, and purification

The Aβ protofibril selective monoclonal antibody mAb158 [3, 27], was recombinantly expressed on a mouse IgG2c backbone, either in regular IgG format or as a bispecific antibody, conjugated to single chain variable fragments of the TfR binding antibody 8D3 [28] (scFv8D3) at the light chain C-termini as previously described [16, 29], resulting in RmAb158-scFv8D3. In an attempt to avoid immunological reactions from repeated antibody injections, RmAb158-scFv8D3 was mutated in its linker and scFv8D3 domain, which is originally from rat, using an online tool for prediction of major histocompatibility complex (MHC) class II (http://tools.iedb.org/mhcii/) binding affinities of T cell epitopes [30]. Next, peptide sequences from MHC II prediction were compared to the mouse genome in Ensembl (https://www.ensembl.org/index.html). Since the thymus is involved in self-tolerance and negatively select for mouse-derived sequences, only sequences that differed from the mouse genome were selected for mutation. Combining the above-described methods resulted in three antibody mutants, RmAb158-scFv8D3^mut 1–3^, that were mutated at different positions in the linker and the scFv8D3 domain (Fig. S1).

A chimeric variant of 8D3 was expressed on the same mouse IgG2c backbone and a scFv of the Aβ antibody 3D6 was fused with scFv8D3 into the small tandem di-scFv3D6-8D3 construct [31]. All antibodies were purified with affinity chromatography (protein G for IgG-based antibodies and immobilized metal affinity chromatography (IMAC) for the tandem di-scFv construct). Size exclusion chromatography purification was used to reduce aggregates of RmAb158-scFv8D3. Anti-CD4 antibody GK1.5 (InVivoMab; Nordic BioSite, Täby, Sweden) was used for depletion of CD4^+^ T cells.

### Treatment studies

#### Acute study

*App*^*NL−G−F*^ mice, 5 months old, were divided into three treatment groups receiving a single intravenous (i.v.) therapeutic dose (32 nmol/kg body weight) of RmAb158-scFv8D3 (*n* = 6) or RmAb158 (*n* = 6), with PBS injection as control (*n* = 5). Brains were extracted 3 days after antibody injection.

#### Inhibition of Aβ seeding

*App*^*NL−G−F*^ mice, 3 months old, were injected intraperitoneally (i.p.) three times every second day with therapeutic doses (32 nmol/kg body weight) of RmAb158-scFv8D3 (*n* = 8) or RmAb158 (*n* = 7) with PBS injection as control (*n* = 9). Repeated injections were done to account for the short half-life of RmAb158-scFv8D3 [19] and to avoid saturation of TfR at the BBB [32]. Brains were isolated after 10 weeks. An additional 3-month-old *App*^*NL−G−F*^ baseline group (*n* = 5) was included to compare with treatment groups.

#### Response to repeated injections

To assess ADA formation after repeated injections, a low therapeutic dose of RmAb158-scFv8D3 (1 mg/kg body weight, corresponding to 5 nmol/kg) was administered to wt mice (3–5 mice per group) with 1–8-week intervals. Each dose was supplemented with a tracer dose (0.1 mg/kg body weight) of radiolabeled RmAb158-scFv8D. Finally, a tracer dose of radiolabeled antibody (0.1 mg/kg body weight) was administered 1 week after the last therapeutic dose to track antibody concentrations in blood, brain, and peripheral organs by radioactivity measurements.

#### ADA prevention studies

Four-month-old wt mice (3–5 mice per group) were immunized with different antibody constructs, modified in different ways to prevent ADA formation. When used as immunogen, all antibodies were dosed by mass. Two i.p. injections were administered, 8 weeks apart, of a low therapeutic dose (1 mg/kg body weight, corresponding to 5 nmol/kg for bispecific IgG; 6.5 nmol/kg for IgG; and 17 nmol/kg for tandem di-scFv). Two weeks later, a diagnostic tracer dose (0.1 mg/kg body weight) of the same antibody, labeled with ^125^I, was administered. Animals were euthanized 1 h post injection and concentration of radiolabeled antibody was measured in blood, brain, and peripheral organs by radioactivity measurements and PET imaging. At this time point, no Aβ related retention of the antibody occurs in the brain. Therefore, wt could be used instead of *App*^*NL−G−F*^ mice to assess antibody biodistribution.

#### CD4^+^ T cell depletion with subsequent chronic anti-Aβ immunotherapy

One day prior to anti-Aβ immunotherapy, 7-month-old *App*^*NL−G−F*^ mice (*n* = 20) were depleted of CD4^+^ T cells with i.p. injection of anti-mouse CD4 monoclonal antibody GK1.5 (dose: 167 mg/kg body weight). After CD4^+^ T cell depletion, mice received weekly i.p. injections for 8 weeks with RmAb158-scFv8D3 (32 nmol/kg body weight, *n* = 5; or 6.4 nmol/kg body weight, *n* = 5); RmAb158 (32 nmol/kg body weight, *n* = 5); or PBS (*n* = 5). Immunotherapy was followed by a diagnostic phase in which all treatment groups received i.v. injection of [^125^I]RmAb158-scFv8D3 (0.6 nmol/kg body weight, 66 MBq/kg). Blood and plasma were collected 1 h, 4 h, 24 h, and 48 h post-injection. Blood, plasma, brain (cortex, hippocampus, and cerebellum), and peripheral organs liver, spleen, and thyroid were extracted 3 days after [^125^I]RmAb158-scFv8D3 injection.

### Brain tissue extraction

Brains were homogenized at 1:10 weight/volume ratio. Tris-buffered saline (TBS) and 70% formic acid (FA) were used to isolate soluble and total Aβ, respectively. Briefly, TBS-homogenized brains were centrifuged for 1 h at 16,000 × *g*, the supernatant was collected and divided into two separate tubes, in which one underwent an additional 100,000 × *g* centrifugation for 1 h. Pellets from TBS homogenization were subsequently dissolved in 70% FA and centrifuged at 16,000 × *g* for 1 h and supernatant was collected.

### Aβ and sTREM2 ELISA quantification

Soluble and membrane-associated Aβ aggregates were analyzed in brain TBS extracts using a sandwich ELISA based on the Aβ N-terminal-specific antibody 3D6 (produced in-house) as both capture and detection antibody. This ELISA will detect Aβ aggregates from the size of a dimer and larger [33]. 96-well half-area plates were coated with 3D6, 50 ng per well, and incubated overnight at + 4 °C. Plates were blocked for 2 h with 1% BSA and brain extracts were added and incubated overnight at + 4 °C. Biotinylated 3D6 was incubated for 2 h at room temperature followed by 1 h of streptavidin-HRP (Mabtech, Nacka Strand, Sweden) at room temperature. ELISA was developed with K-blue Aqueous TMB substrate (Neogen Corp., Lexington, KY, USA) and read with a spectrophotometer at 450 nm. Samples were quantified against a calibration standard curve of Aβ protofibrils, prepared by size exclusion chromatography purification of Aβ1-42 (Innovagen, Lund, Sweden) after 3 h incubation at 37 °C.

Total Aβ1–40 and 1–42 were analyzed in formic acid soluble brain extracts using anti-Aβ40 (custom production, Agrisera, Umeå, Sweden) and anti-Aβ42 (ThermoScientific, Waltham, MA, US) antibodies. Briefly, 96-well plates were coated overnight at + 4 °C with 50 ng anti-Aβ40 or anti-Aβ42 antibody, then blocked with 1% BSA for 2 h at room temperature. FA brain samples were neutralized with 2 M tris buffer and incubated overnight at + 4 °C, then detected with biotinylated 3D6 and streptavidin-HRP as above.

Levels of soluble triggering receptors expressed on myeloid cells 2 (sTREM2) in TBS brain extracts were analyzed with a sandwich ELISA, as previously described [34]. 96-well were coated with AF1729 (R&D, Abingdon, UK) (20 ng per well) overnight at 4 °C, then blocked with 1% BSA. Brain extracts were diluted 80 times, incubated overnight at 4 °C, and detected with a 2 h incubation of biotinylated BAF1729 (R&D), followed by streptavidin-HRP conjugate (Mabtech AB) for 1 h. Signals were developed and read as above.

### Immunofluorescence

Brain tissue cryosections (20 µm) from CD4^+^ T cell depleted, antibody-treated *App*^*NL−G−F*^ mice were immunostained to visualize Aβ42 or total Aβ and the microglial marker ionized calcium-binding adaptor molecule 1 (Iba-1). Sections were fixed in 4% paraformaldehyde (PFA), followed by antigen retrieval in preheated citrate buffer (25 mM, pH 6.0). After equilibration to room temperature, sections were treated with 70% formic acid for 5 min to promote Aβ immunoreactivity, then permeabilized with 0.4% Triton-X 100 in PBS and blocked with 5% normal goat serum (Bionordika, Solna, Sweden). Sections were incubated overnight at + 4 °C with primary antibodies (anti-Aβ42, 1:500; Invitrogen), (6E10, 1:300; Nordic Biosite, Täby, Sweden) and (anti-Iba1, 1:450; ab178846, Abcam, Cambridge, UK) in 0.1% Tween in PBS, then for 1 h with secondary antibodies; goat anti-rabbit (1:500 Alexa fluor 488, A11008, Sigma-Aldrich), goat anti-mouse (1:500 Alexa fluor 488, A11029, Sigma-Aldrich) and goat anti-rabbit (1:500 Alexa fluor 594, A11037 Sigma-Aldrich) in 0.1% Tween20 in PBS, followed by mounting with Vectashield® Antifade Mounting Medium with DAPI (Vector Laboratories). Immunofluorescence was imaged with a Zeiss Observer Z.1 microscope and ZEN 2.6 software (Carl Zeiss Microimaging GmbH, Jena, Germany) and quantified with Fiji Image J [35]. Aβ42 staining was quantified as the percentage of the total measured area and as the integrated density (mean intensity × area) for five cortical and two hippocampal regions of interest. M.O.M ® Immunodetection Kit, basic (Vector Laboratories, Burlingame, CA, USA) were used on sections stained with 6E10 according to the manufacturer’s instructions.

### Radiochemistry

Antibodies were labeled with iodine-125 (^125^I) for single photon emission computed tomography (SPECT) and ex vivo experiments and with iodine-124 (^124^I) for positron emission tomography (PET) experiments, using direct iodination with chloramine-T [36]. Briefly, for ^125^I labeling, antibodies, ^125^I stock solution (Perkin-Elmer), and 5 μg Chloramine-T (Sigma-Aldrich, Stockholm, Sweden) were mixed in PBS with a final volume of 110 μl. Labeling reactions were quenched with 10 μg sodium metabisulfite (Sigma-Aldrich) after 90 s. For ^124^I labeling, 30 MBq ^124^I was incubated for 15 min with 50 μM NaI, followed by the addition of 34 μg RmAb158-scFv8D3 and 20 μg Chloramine-T to a final volume of 210 μl. The reaction was quenched after 120 s with 40 μg sodium metabisulfite. Radioiodinated proteins were purified with NAP-5 size exclusion columns (GE healthcare AB, Uppsala, Sweden) and eluted in 700 μl PBS.

### PET/SPECT imaging

PET or SPECT was used to visualize biodistribution of radiolabeled antibodies. Mice were anesthetized with 1.5 − 2.0% isoflurane and placed in a Mediso small animal PET or SPECT scanner, depending on the study. Acquisition time was 60 min, followed by a 3-min CT examination with a 9.8-cm field of view. PET data were reconstructed using an OSEM 3D algorithm (20 iterations). SPECT acquisition data were reconstructed using Nuclide 2.03 software and Tera-Tomo™ 3D SPECT re-constructive algorithm (Mediso Medical Imaging Systems, Hungary) with scattering and attenuation correction. The CT raw files were reconstructed using filtered back projection. All subsequent processing of the PET, SPECT, and CT images was performed in the imaging software Amide 1.0.4.

### Biodistribution and gamma counter measurement

At the study endpoint, mice were anesthetized with 3% isoflurane, a terminal blood sample was obtained from the heart before mice were transcardially perfused with 0.9% saline. Plasma was obtained by centrifugation of the terminal blood sample. Brains were extracted and hemispheres were separated. One hemisphere was frozen and analyzed by autoradiography, while the other hemisphere was dissected into the cortex, hippocampus, and cerebellum. The thyroid, spleen, and liver were also isolated from mice that underwent CD4 immunodepletion. Tissue radioactivity was measured in a gamma counter (Wizard™, Wallac Oy, Turku, Finland), and radioactivity concentration in the tissue was quantified as percent injected dose per gram tissue (%ID/g).

### Autoradiography

Frozen sagittal brains were sectioned into 20 μm cryosections and exposed to a phosphor imager plate for 2 weeks, with a standard of known radioactivity. The exposed plate was read at a resolution of 20 μm in a typhoon phosphor imager scanner (GE). Fiji (v1.8.0) in combination with royal lookup table was used to visualize antibody retention in brain.

### Anti-drug antibody ELISA

The presence of plasma ADA in immunized mice was detected with a bridging ELISA, in which coating and biotinylated antibodies were the same as the treatment antibody to which an immune response would be directed. Briefly, 96-well plates were coated with 5 nM of antibody and incubated overnight at + 4ºC. Wells were blocked for 2 h with 1% BSA blocking buffer, and plasma samples in 0.1% BSA (1:100) were loaded and incubated overnight at + 4 °C. Five nM detection antibody (biotinylated version of the same antibody used for coating) was added and incubated for 1 h at room temperature, followed by 1 h of incubation with streptavidin-HRP, then developed as above.

### Statistical analyses

Statistical analyses were performed in GraphPad Prism 9.0.0 (GraphPad Software, Inc., San Diego, CA). Results are reported as mean ± standard deviation. Statistical assessment was carried out by multiple unpaired *t*-test or one-way ANOVA with Tukey’s multiple comparisons test. * *p* < 0.05; ** *p* < 0.01; *** *p* < 0.001.

## Results

### “Disease progression” in *App*^*NL−G−F*^ mice

**Fig. 1:**
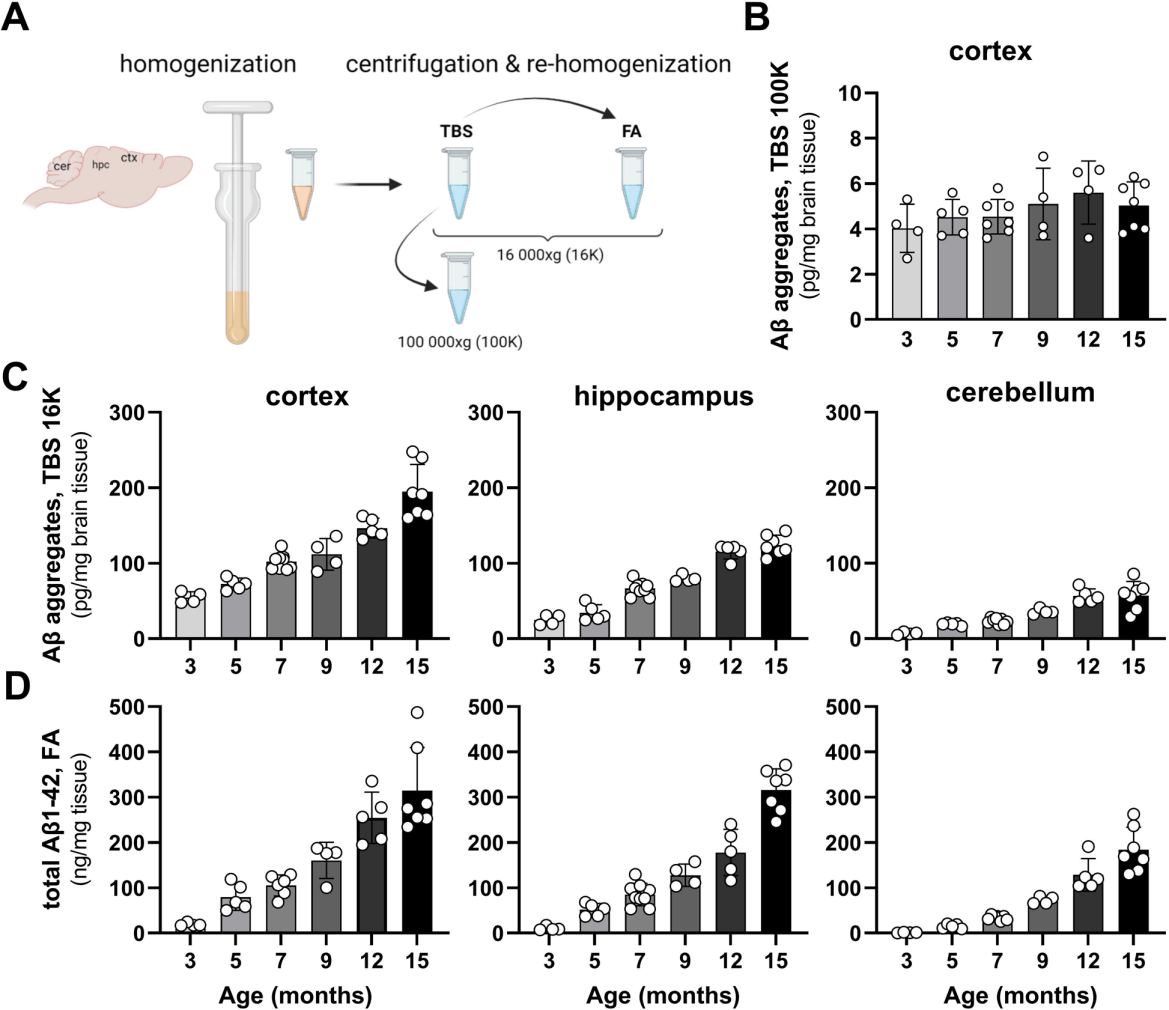
Development of Aβ pathology in *App*^*NL−G−F*^ mice over time. **A** Schematic image of sequential brain extraction. **B** ELISA quantification of soluble Aβ aggregates in TBS brain (cortex) extract centrifuged at 100,000 × *g* (TBS 100 K) from *App*^*NL−G−F*^ mice of different ages. **C**. ELISA quantification of Aβ aggregates in TBS brain extracts centrifuged at 16,000 × *g* (TBS 16 K) and of Aβ1-42 in FA extracts **D** of the cortex, hippocampus, and cerebellum of *App*^*NL−G−F*^ mice at different ages. **A** was created with BioRender

Brain regions (cortex, hippocampus, and cerebellum) from *App*^*NL−G−F*^ mice of different ages, were sequentially homogenized (Fig. 1A) and analyzed with ELISA to quantify how pools of Aβ change over time. The most soluble Aβ aggregates, retrieved from cortex TBS extract centrifuged at 100,000 × *g* (TBS 100 K) and measured with a 3D6 sandwich ELISA, appeared at low and relatively constant levels with little change over time (Fig. 1B). In contrast, TBS extracts centrifuged at a lower speed, 16,000 × *g* (TBS 16 K), showed a gradual increase of Aβ over time, starting from 3 months of age and still increasing up to the age of fifteen months. Although levels were different, a similar pattern was seen in all three brain regions (Fig. 1C). FA soluble Aβ, representing total Aβ1-42 and thereby a measure of Aβ plaque burden, started to appear at low concentration in the cortex of 3-month-old *App*^*NL−G−F*^ mice and then increased with age (Fig. 1D). Again, a similar pattern was seen in hippocampus and cerebellum, but with a somewhat later age of onset.

### Anti-Aβ immunotherapy in *App*^*NL−G−F*^ mice

**Fig. 2:**
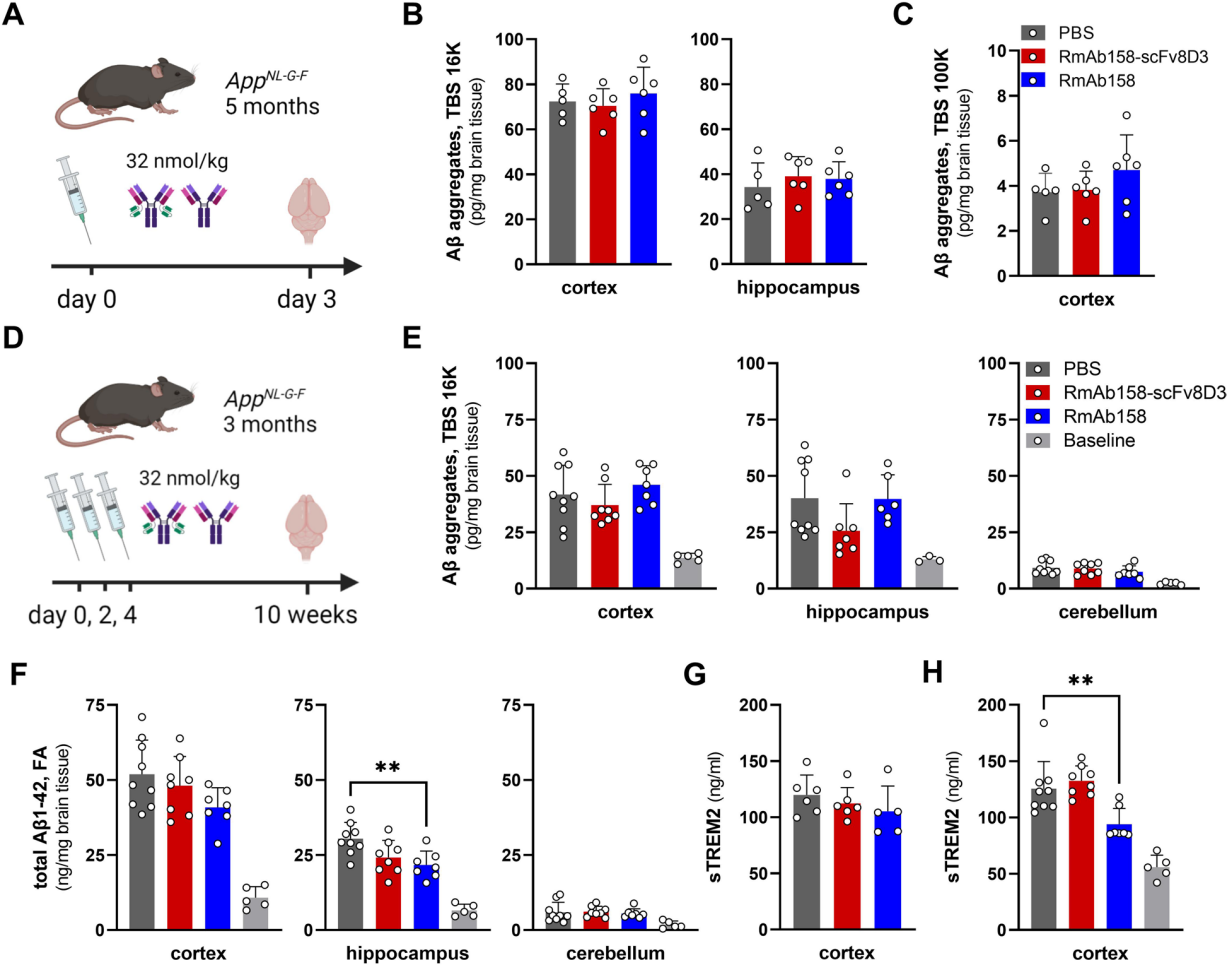
Effects of acute antibody treatment in *App*^*NL−G−F*^ mice. **A** Study design: 5-month-old *App*^*NL−G−F*^ mice were treated with a single injection of PBS, RmAb158, or RmAb158-scFv8D3 (32 nmol/kg body weight). Brains were isolated after 3 days to analyze brain levels of Aβ. Quantification of soluble Aβ aggregates in TBS 16 K brain extracts of cortex and hippocampus (**B**) and in cortical TBS 100 K extracts (**C**). **D** Study design: 3-month-old *App*^*NL−G−F*^ mice were treated with three single injections of PBS, RmAb158, or RmAb158-scFv8D3 (32 nmol/kg body weight) during one week. Brains of treated mice, and a baseline group of non-treated 3-month-old mice, were isolated ten weeks later to analyze brain levels of Aβ. Quantification of Aβ aggregates in TBS 16 K brain extracts (**E**) and of Aβ1–42 in FA soluble extracts of the cortex, hippocampus, and cerebellum (**F**). ELISA quantification of soluble TREM2 (sTREM2) in TBS 16 K extracts from the acute (**G**) and seeding-inhibition (**H**) studies. Values represent mean and SD. Note that the unit in **B**, **C**, and **E** (pg/mg brain) differs from that in **F** (ng/mg brain). **A** and **D** were created with BioRender

The effect of RmAb158-scFv8D3 and RmAb158 on soluble Aβ aggregates was studied in 5-month-old *App*^*NL−G−F*^ mice 3 days after a single dose of antibody (32 nmol/kg body weight) (Fig. 2A). Neither of the antibodies reduced Aβ aggregates in TBS 16 K extract of cortex and hippocampus (Fig. 2 B). However, in this acute therapeutic setting, where the antibody was administered only 3 days before assessment of Aβ pathology, a reduction would most likely be seen only in the most soluble pool of Aβ aggregates, i.e., in the TBS 100 K extract, that was subjected to centrifugation at 100,000 × *g*. Yet, unlike previous studies, no treatment effect was seen with either of the antibodies in *App*^*NL−G−F*^ mice (Fig. 2C).

Targeting the earliest formation of Aβ aggregates could inhibit seeding and further aggregation and deposition of Aβ in the brain. Thus, 3-month-old *App*^*NL−G−F*^ mice, where Aβ pathology is emerging, were administered three doses of RmAb158-scFv8D3 or RmAb158 during 1 week to clear the brain from these first Aβ aggregates (Fig. 2D). Ten weeks after antibody treatment, no significant effect was seen on levels of TBS soluble Aβ aggregates (Fig. 2E). However, total Aβ1-42 was significantly reduced in the hippocampus of RmAb158 treated mice, with a similar trend in the RmAb158-scFv8D3 group (*p* = 0.066). A trend was also seen in the cortex of RmAb158-treated mice, whereas no effects were seen in the cerebellum. We have previously observed that brain levels of soluble TREM2 follow Aβ pathology [34] and are reduced by Aβ lowering treatment [18, 34]. Here, we did not see any effect on sTREM2 in cortical TBS 16 K extract 3 days after acute treatment (Fig. 2G). However, ten weeks after treatment, there was a significant reduction of sTREM2 in the cortex of mice treated with RmAb158 (Fig. 2H), probably reflecting a general lowering of Aβ pathology.

### Impact of repeated injections

**Fig. 3:**
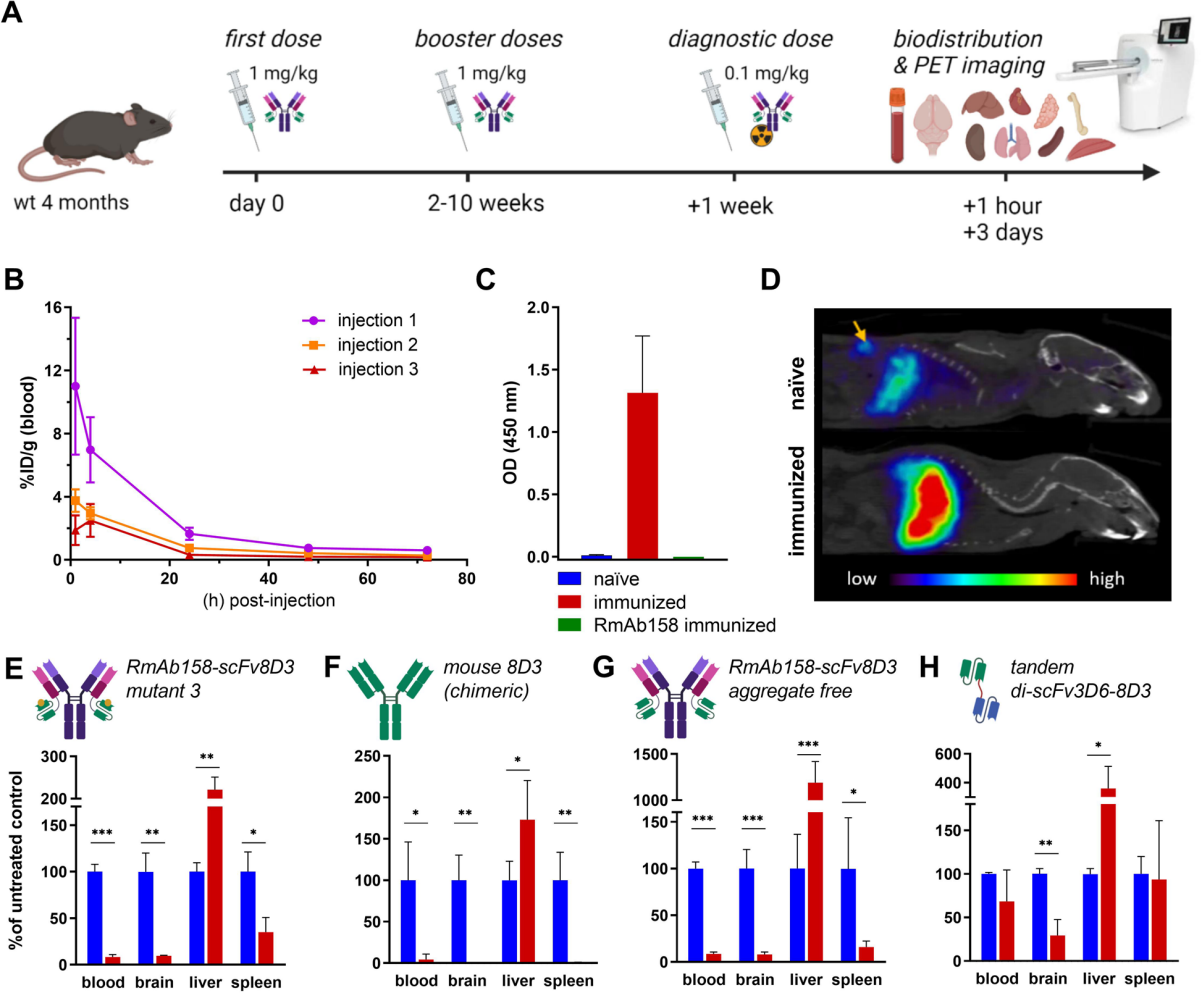
Effect of repeated antibody injections in wt mice. **A** Study design: 4-month-old wt mice, subjected to repeated antibody injections, were injected with an iodinated variant of the same antibody (diagnostic dose), followed by extraction of blood, brain, and organs. **B** Blood pharmacokinetcs after 1^st^, 2^nd^, and 3^rd^ injection of [^125^I]RmAb158-scFv8D3 in wt mice. **C** ELISA measuring ADA in plasma from mice in **B** in comparison with naïve (untreated) and RmAb158 treated mice, using RmAb158-scFv8D3 as both capture and detecting antibody. **D** Sagittal half-body PET images obtained during 0–10 min after injection of [^124^I]RmAb158-scFv8D3^mut 3^ in wt mice with or without pretreatment with the same antibody. Yellow arrow points to the spleen, which was readily visible in naïve control mouse. Blood, brain, liver, and spleen radioactivity concentration 1 h after injection of radiolabeled RmAb158-scFv8D3^mut 3^ (**E**), mouse chimeric 8D3 IgG (**F**), aggregate free RmAb158-scFv8D3 (**G**), or tandem di-scFv3D6-8D3 (**H**) in untreated mice (blue) or after repeated injections of the same antibody (red). **A** was created with BioRender

To assess the impact of repeated injections of bispecific RmAb158-scFv8D3, which contains protein domains from foreign species (8D3 was raised in rat), 4-month-old wt mice were subjected to repeated injections of RmAb158-scFv8D (Fig. 3A). Mice displayed an immediate reduction in blood exposure of [^125^I]RmAb158-scFv8D3 already at the second dose (Fig. 3B). The presence of a strong ADA response in plasma from immunized animals was confirmed with a sandwich ELISA assay using the therapeutic antibody as both capture and detection antibody. No such response was seen in plasma from naïve animals or from animals that received repeated injections of RmAb158 (Fig. 3C).

To reduce immunogenicity of the antibody, the scFv8D3 amino acid sequence was mutated at various positions to reduce interaction with MHC class II. Three mutated antibodies, RmAb158-scFv8D3^mut 1–3^ (Fig. S1), were repeatedly injected in wt mice to provoke an immune response. Indeed, ADA formation seemed to be reduced in [^124^I]RmAb158-scFv8D3^mut 3^ injected mice (Fig. S2). To assess its biodistribution in real-time, the antibody was radiolabeled with ^124^I for PET imaging. Already 10 min after injection, [^124^I]RmAb158-scFv8D3^mut 3^ had accumulated in the liver of mice pre-treated with RmAb158-scFv8D3^mut 3^. The untreated control mouse displayed lower liver uptake, whereas spleen uptake, characteristic of in vivo TfR interaction, was clearly visible (Fig. 3D, Video S1). Ex vivo analysis showed that both blood, brain, and spleen retention of the antibody was markedly reduced 60 min after injection. In contrast, and confirming PET imaging results, liver retention of the antibody was increased (Fig. 3E), suggesting it was degraded in the liver. Since immunogenicity-reducing mutations in scFv8D3 did not prevent the antibody from degradation, the variable domains of 8D3 were cloned into a mouse IgG2c. This mouse chimeric 8D3 antibody was assumed to be less prone to elicit immune responses in the mouse since it lacked the unnatural linkers in the scFv, although it still contained variable domains from rat. However, similar to previous experiments, blood, brain and spleen were completely depleted from antibody, while liver concentration increased (Fig. 3F). Thus, since no improvement was achieved with a murinized version of the 8D3 antibody, there could be a reason to suspect that other mechanisms might be involved, such as in vivo aggregation or Fc mediated cellular effects. To assess these options, an aggregate free preparation of RmAb158-scFv8D3 as well as the small bispecific antibody construct tandem di-scFv3D6-8D3, which lacks an Fc domain were injected following the same scheme. While the aggregate free RmAb158-scFv8D3 showed no improvement (Fig. 3G), di-scFv3D6-8D3 was less affected by repeated injections, as it displayed a non-significant decrease in both blood and spleen and a more modest reduction in brain concentration compared with the other antibody constructs, but still with a significant increase in liver uptake, indicating some degradation (Fig. 3H).

**Fig. 4:**
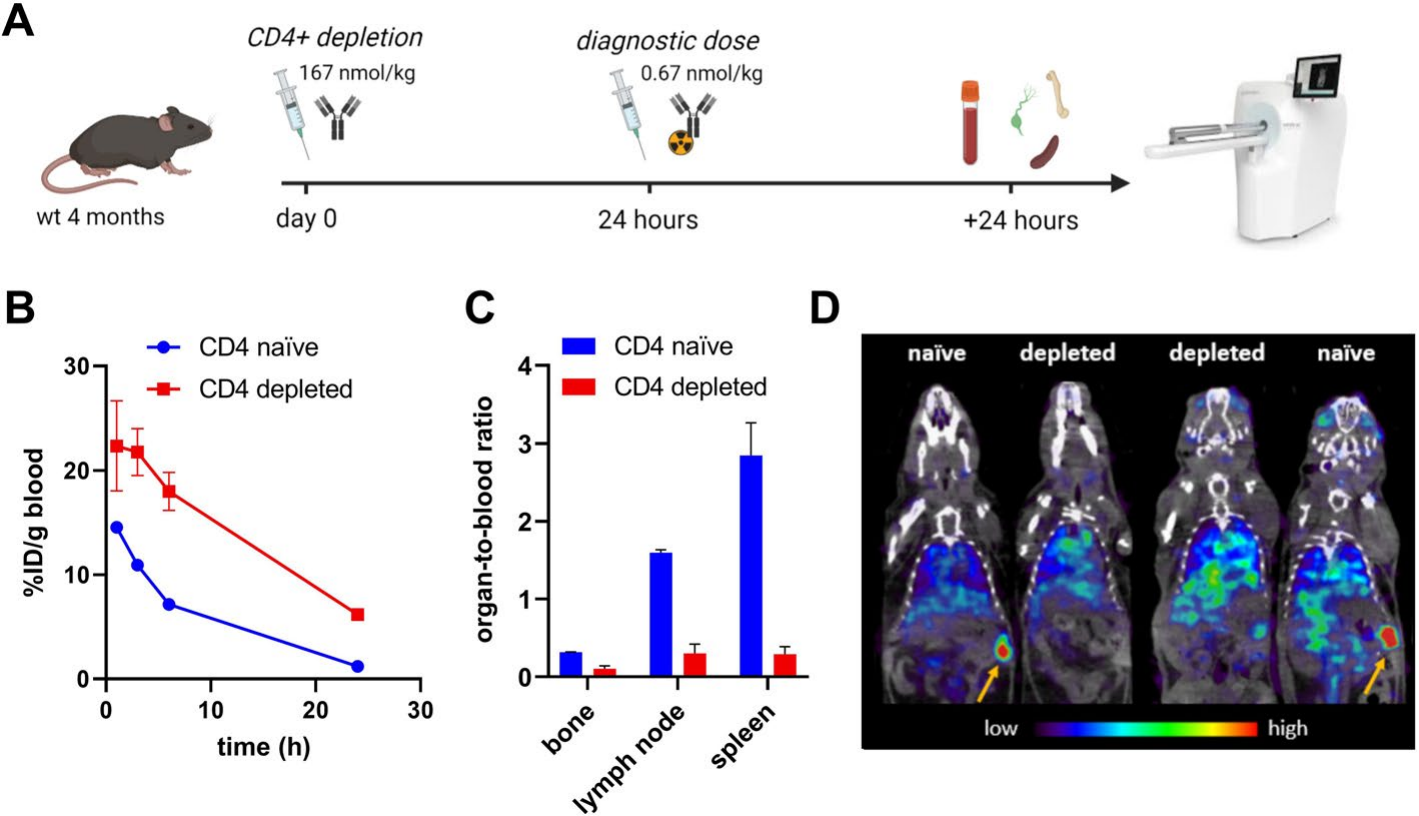
CD4^+^ T cell depletion in wt mice. **A**. Study design: 4-month-old wt mice were injected with anti-CD4 antibody GK1.5 at 167 nmol/kg body weight to deplete them from CD4^+^ T cells. After 24 h, a tracer dose of [^125^I]GK1.5 was injected in CD4^+^ T cell depleted and naïve mice to image any remaining CD4^+^ T cells. Blood pharmacokinetics (**B**), distribution to organs (**C**), and SPECT imaging (**D**) of [^125^I]GK1.5 in CD4^+^ T cell depleted (*n* = 2) and naïve (*n* = 2) wt mice 24 h after injection.** A** was created with BioRender

To circumvent ADA-mediated reduction in plasma concentrations of functional antibody, depletion of CD4^+^ T cells was explored. First, wt mice were given a single dose of the anti-CD4 antibody GK1.5 to deplete them of CD4^+^ T cells, which are crucial for formation of an antibody response. The next day, mice were injected with radiolabeled GK1.5 to track its pharmacokinetics and biodistribution by radioactivity measurements and SPECT imaging (Fig. 4A). Mice depleted of CD4^+^ T cells displayed higher blood concentrations of [^125^I]GK1.5 (Fig. 4B), suggesting less binding to CD4 in the tissues. This was also evident from a much lower relative distribution to bone (marrow), lymph nodes, and spleen (Fig. 4C), organs normally containing CD4^+^ T cells. SPECT imaging clearly showed a high uptake of [^125^I]GK1.5 in the spleen of naïve mice, while this signal was lacking in CD4-depleted mice (Fig. 4D). Taken together, these results suggest that depletion of CD4^+^ T cells was effective.

### Effects of chronic anti-Aβ therapy

**Fig. 5:**
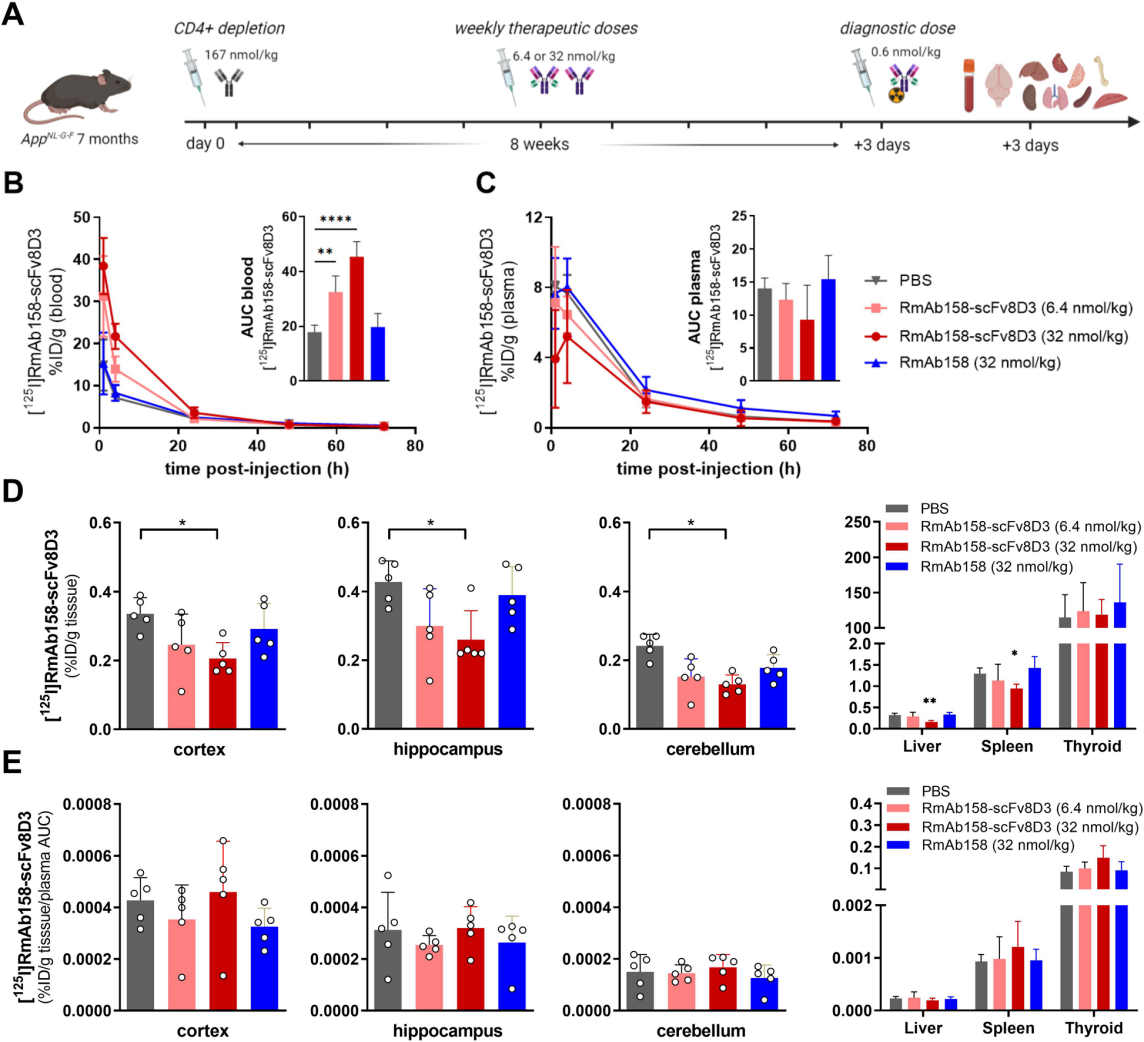
Pharmacokinetics and biodistribution of [^125^I]RmAb158-scFv8D3 following chronic antibody treatment.** A** Study design: 7-month-old *App*^*NL−G−F*^ mice were depleted of CD4^+^ T cells by injection of anti-CD4 antibody (167 nmol/kg), followed by weekly i.p. injections of RmAb158-scFv8D3 (6.4 or 32 nmol/kg) or RmAb158 (32 nmol/kg) during 8 weeks. Three days after the last injection, a diagnostic tracer dose (0.6 nmol/kg) of [^125^I]RmAb158-scFv8D3 was administered i.v. Blood (**B**) and plasma (**C**) pharmacokinetics of [^125^I]RmAb158-scFv8D3 with inserts displaying total exposure over 72 h (area under the curve – AUC). **D** [^125^I]RmAb158-scFv8D3 retention in the cortex, hippocampus, cerebellum, and peripheral organs liver, spleen, and thyroid. **E** [^125^I]RmAb158-scFv8D3 concentration, individually adjusted for total exposure (plasma AUC) in the cortex, hippocampus, cerebellum, and peripheral organs liver, spleen, and thyroid. Values are mean and SD. **A** was created with BioRender

*App*^*NL−G−F*^ mice were CD4^+^ T cell immunodepleted to facilitate subsequent chronic immunotherapy with the bispecific antibody, RmAb158-scFv8D3. Mice received nine weekly i.p. injections and a final diagnostic i.v. injection of radioiodinated RmAb158-scFv8D3 (Fig. 5A). A high treatment dose of RmAb158-scFv8D3 was associated with enhanced blood retention of [^125^I]RmAb158-scFv8D3 (Fig. 5B). However, in isolated blood plasma, there was an inverse relation in which high treatment dose of RmAb158-scFv8D3 resulted in a trend towards lower free plasma concentration of [^125^I]RmAb158-scFv8D3 (Fig. 5C). Brain retention of the antibody followed the same pattern, with significantly lower [^125^I]RmAb158-scFv8D3 concentrations in all brain regions of *App*^*NL−G−F*^ mice administered with the high dose of RmAb158-scFv8D3 (Fig. 5D). Also in the periphery, [^125^I]RmAb158-scFv8D3 displayed a similar distribution, with low retention in liver and spleen in the high dose RmAb158-scFv8D3 treatment group (Fig. 5D). When correcting for the exposure of [^125^I]RmAb158-scFv8D3, all significant differences between groups disappeared (Fig. 5E), suggesting that dose dependent changes in blood and plasma concentrations of [^125^I]RmAb158-scFv8D3 affected the antibody’s distribution to all tissues.

**Fig. 6:**
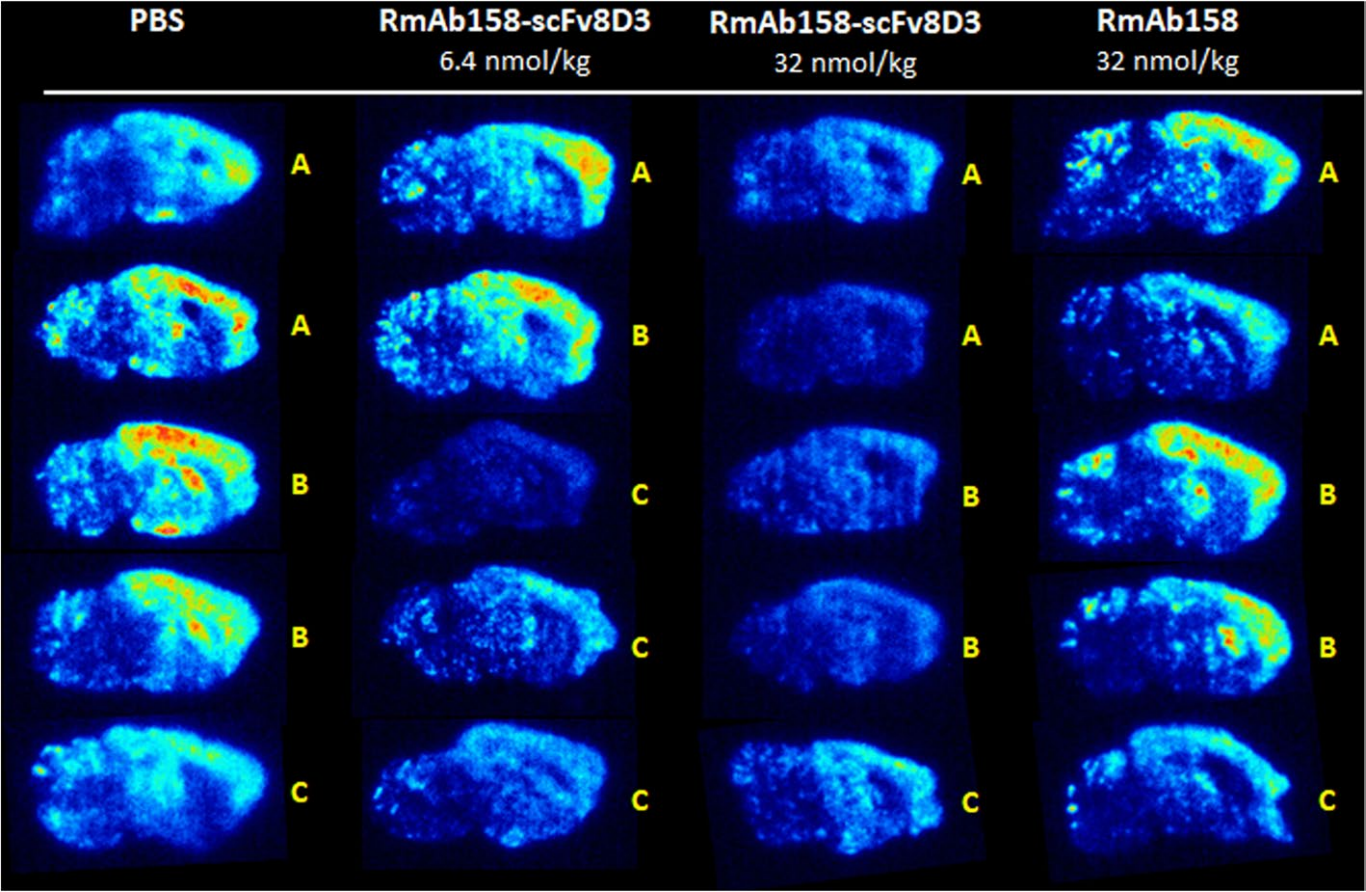
Autoradiography. Ex vivo brain autoradiography after tracer dose of [^125^I]RmAb158-scFv8D3 in CD4^+^ T cell depleted *App*^*NL−G−F*^ mice treated with PBS; low or high dose of RmAb158-scFv8D3; or high dose of RmAb158. The different litters are represented by letters (A–C) to illustrate similarities between individuals of the same litter and differences between different litters

Following CD4^+^ T cell depletion, immunotherapy, and diagnostic administration, sagittal brain sections were exposed to an autoradiography plate to investigate retention and spatial distribution of [^125^I]RmAb158-scFv8D3. Similar to ex vivo quantification of brain retention, mice treated with a high dose of RmAb158-scFv8D3 for 8 weeks displayed the lowest brain retention of [^125^I]RmAb158-scFv8D3, compared with low dose RmAb158-scFv8D3, RmAb158, and PBS treated mice which displayed high retention (Fig. 6). Brain retention of [^125^I]RmAb158-scFv8D3 was mainly observed in brain regions known to harbor Aβ pathology, i.e., cortex, hippocampus, and thalamus, but to some extent also in the cerebellum. Notably, there was a considerable variation between different mouse litters (Fig. 6).

**Fig. 7:**
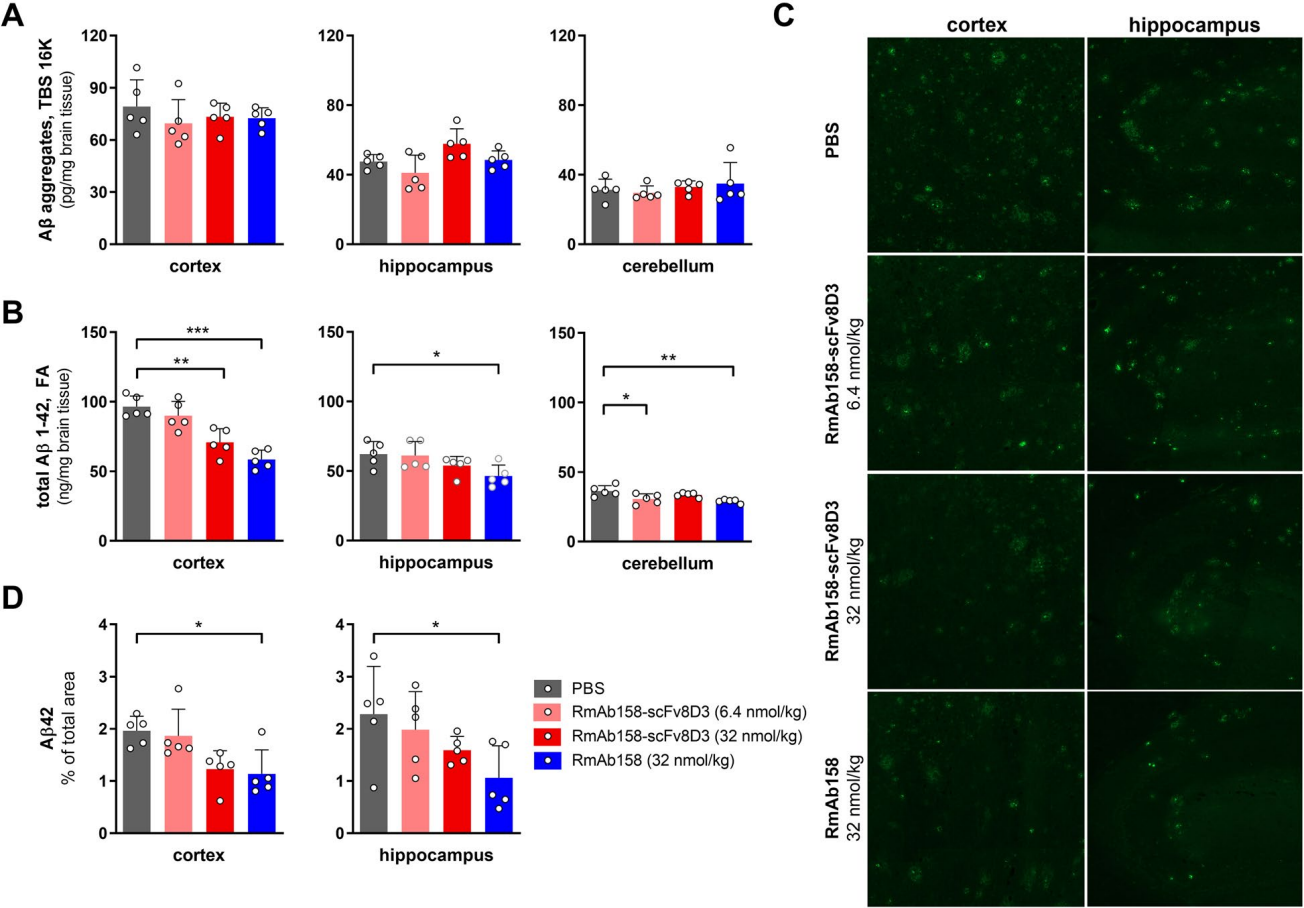
Aβ quantification after chronic antibody treatment. Concentrations of soluble Aβ aggregates in TBS 16 K extracts (**A**) and of total Aβ1–42 in FA soluble extracts (**B**) of cortex, hippocampus, and cerebellum from CD4^+^ T cell depleted *App*^*NL−G−F*^ mice treated with PBS, low (6.4 nmol/kg) or high (32 nmol/kg) dose of RmAb158-scFv8D3, or with RmAb158 (32 nmol/kg). **C** Representative images of Aβ42 immunostaining in the cortex and hippocampus from antibody-treated *App*^*NL−G−F*^ mice. **D** Quantification of Aβ42 immunostaining in the cortex and hippocampus of antibody-treated *App*^*NL−G−F*^ mice

Brain levels of soluble Aβ aggregates in TBS 16 K extracts of all treatment groups were similar in all studied brain regions (Fig. 7A). However, when assessing FA soluble Aβ1-42, accounting for the vast majority of Aβ in the brain, it was evident that treatment had significantly reduced levels of Aβ in all brain regions (Fig. 7B). This reduction was most pronounced in the cortex, where Aβ1-42 levels were reduced in mice treated with a high dose of RmAb158-scFv8D3 (*p* < 0.01) and RmAb158 (*p* < 0.001) compared with PBS. A similar trend was seen in the hippocampus, but a significant reduction was only observed in RmAb158 compared with PBS-treated mice (*p* < 0.05). Cerebellar Aβ1–42 displayed a significant reduction in mice treated with RmAb158 and low dose RmAb158-scFv8D3 compared with PBS (*p* < 0.05). These results were largely confirmed by Aβ42 immunostaining that showed a significant reduction in both cortex and hippocampus of RmAb158 compared with PBS-treated mice (*p* < 0.05) and a similar trend for RmAb158-scFv8D3 treated mice (Fig. 7C, D; Fig S3).

**Fig. 8:**
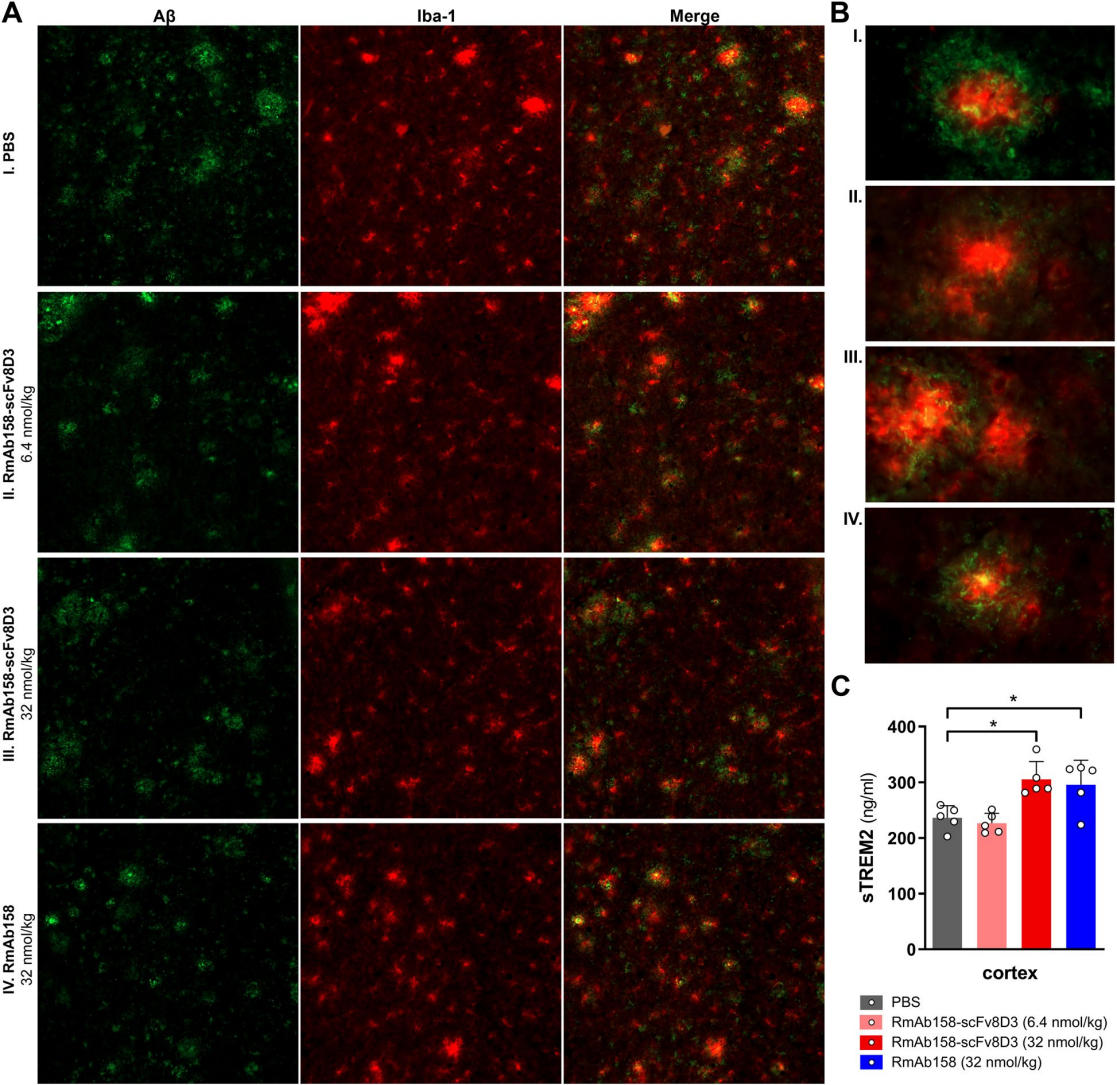
Microglial response to chronic antibody treatment.** A** Immunostaining of Aβ (6E10; green) and microglial marker Iba-1 (red) in cortex from representative CD4^+^ T cell depleted *App*^*NL−G−F*^ mice treated with PBS, low (6.4 nmol/kg) or high (32 nmol/kg) dose of RmAb158-scFv8D3, or with RmAb158 (32 nmol/kg). **B** Merged images of Aβ and Iba-1 staining of individual Aβ deposits in the cortex of the same mice as in **A**. **C** ELISA quantification of sTREM2 in TBS 16 K extracts from the cortex of treated mice. Values are mean and error bars represent standard deviation

Microglia in the cortex of treated mice, assessed with Iba-1 immunostaining, were found in large numbers around Aβ deposits in all groups of mice (Fig. 8A). The highest immunostaining intensity was found associated with the cores of Aβ deposits (Fig. 8B). To assess specific microglial response to treatment, levels of sTREM2 were quantified with ELISA in TBS 16 K brain extracts. As a result of chronic treatment, sTREM2 was increased in the cortex of mice treated with both RmAb158-scFv8D and RmAb158 at a high dose, compared to PBS (*p* < 0.05) (Fig. 8C).

## Discussion

This study compared Aβ lowering abilities of a bispecific BBB penetrating anti-Aβ antibody, RmAb158-scFv8D3, in comparison with its monospecific variant RmAb158. We and others have previously shown that early treatment with mAb158 hinders Aβ plaque formation or reduces Aβ plaque burden [37–39], likely involving glial cell-mediated uptake and clearance [40]. Furthermore, a single dose of its bispecific form, RmAb158-scFv8D3, is as efficient in removing soluble Aβ, at a ten times lower dose compared with monospecific RmAb158 [20]. Similar results have been achieved with a bispecific antibody targeting the protein alpha-synuclein in a transgenic mouse model of Parkinson’s disease [21, 22]. Here we evaluated multiple treatment regimes in the human *APP* knock-in mouse model *App*^*NL−G−F*^, which is characterized by early and aggressive brain Aβ amyloidosis, with parenchymal Aβ depositions from 2 months of age [24]. Unlike our previous results in tg-ArcSwe mice, a single injection of RmAb158-scFv8D3 did not lower soluble Aβ in *App*^*NL−G−F*^ mice. The lack of acute treatment effect in this mouse model is likely due to the fact that, compared with tg-ArcSwe mice, the *App*^*NL−G−F*^ brain contains low concentrations of soluble Aβ aggregates [41], which is the main target of RmAb158 and its bispecific variant. Next, we explored if acute RmAb158-scFv8D3 therapy at an early stage of Aβ deposition could remove Aβ seeds and thus postpone Aβ build-up in the brain. This mechanism has previously been demonstrated in APP23 mice after a short treatment (three doses during a week) with *aducanumab* [42]. Significant reduction of total Aβ1-42 was seen in the hippocampus of *App*^*NL−G−F*^ mice treated with RmAb158 and a similar trend was seen in the RmAb158-scFv8D3 high dose group. Although such trends were seen also in the cortex, no significant reduction was obtained. Interestingly, at the start of this therapeutic intervention, the total Aβ1-42 was already elevated in the cortex, while the hippocampus was still virtually devoid of Aβ pathology. This is in agreement with previous reports and could explain why a delay in pathology seemed to occur primarily in the hippocampus [43]. Further studies, including additional mouse models of Aβ pathology, are needed to elucidate the clinical relevance of these mechanisms for Aβ removal. Soluble Aβ aggregates and fibrillar seeds have been identified as antibody targets in post mortem AD brain tissue [10, 44], but clearance of such Aβ species has never been assessed in clinical trials of AD immunotherapy. However, interesting information about aspects of early intervention can be expected from future clinical trials in patients at early stages of Aβ pathology. Such trials, where treatment starts in mild or asymptomatic patients, will also demonstrate whether an even earlier intervention will lead to more substantial clinical benefit, with a more robust slowing or delay of cognitive decline.

Treatment with biological therapeutics, based on antibodies and other large macromolecules with complex structures, has given rise to potentially detrimental immunological responses such as infusion reactions [45] and humoral responses as ADA [46]. Development of fully human antibodies or humanization of antibodies has mitigated the risk of ADA reactions in the current generation of immunotherapeutic antibodies. RmAb158-scFv8D3 is based on a mouse heavy and light chain IgG with rat scFv connected to the C-terminus of the RmAb158 light chain with peptide linker [16]. Here, repeated injections of RmAb158-scFv8D3 resulted in altered blood kinetics with rapid liver accumulation only minutes after injection, accompanied by low blood concentration, leading to severely reduced brain concentrations of RmAb158-scFv8D3. Bridging ELISA showed an ADA response directed towards RmAb158-scFv8D3, but not to RmAb158, following multiple injections over several weeks. Mutations introduced to reduce scFv8D3 immunogenicity did have a modest effect in reducing ADA formation, but the antibody remained severely affected by reduced blood concentration and brain uptake. Protein aggregation can promote B cell receptor clustering resulting in B cell activation and humoral response [47]. Still, administration of an aggregation-free formulation of RmAb158-scFv8D3 did not reduce ADA formation, and blood retention and brain uptake remained low. Also, a mouse chimeric 8D3 antibody gave rise to an ADA response, resulting in lowered blood retention and brain uptake, despite its fully murinized constant domains and lack of foreign linkers. Assessment of a bispecific format that lacked an Fc region and had a monovalent mTfR binding mode decreased the impact of multiple injections on blood pharmacokinetics significantly. Brain uptake was still reduced, but to a lesser degree. Although liver uptake was high, indicating hepatic degradation, spleen concentration was not affected by repated injections of the Fc region lacking di-cFv3D6-8D3 construct. These results suggest that multiple different mechanisms may contribute to the alteration of antibody pharmacokinetics after repeated injections. For example, Fc mediated interactions with immune cells in the blood may be initiated by bivalent mTfR interaction of RmAb158-scFv8D3, which after repeated injections trigger an immune dependent degradation of the antibody in the liver.

To attenuate immune response towards, and facilitate repeated injections of RmAb158-scFv8D3, *App*^*NL−G−F*^ mice were subjected to CD4^+^ T cell depletion. A diagnostic dose of [^125^I]RmAb158-scFv8D3 was administered i.v. after 8 weeks of chronic treatment to assess whether antibody brain retention could be used to quantify the Aβ-targeted treatment effect, as previously demonstrated with [^124^I]RmAb158-scFv8D3 immunoPET imaging [17, 18]. Analysis of [^125^I]RmAb158-scFv8D3 concentration in brain and blood revealed no correlation with treatment effect, but a dose-dependent alteration in blood and plasma in both groups that received RmAb158-scFv8D3 therapy. The higher blood concentration and lower plasma concentration of [^124^I]RmAb158-scFv8D3 in mice treated with bispecific RmAb158-scFv8D3 was clearly associated with a reduced brain uptake. Although the total brain uptake of [^125^I]RmAb158-scFv8D3 was clearly affected by treatment, ex vivo autoradiography displaying the spatial brain distribution of [^125^I]RmAb158-scFv8D3 showed that the antibody was accumulated in brain regions overlapping with those known to harbor Aβ pathology in *App*^*NL−G−F*^ mice. Thus, BBB penetration of [^125^I]RmAb158-scFv8D3 was only partly reduced. Biochemical and histological analysis of Aβ pathology revealed a comparable treatment effect in lowering cortical Aβ42 in animals receiving chronic treatment with RmAb158-scFv8D3 and RmAb158. Immunostainings showed abundant microglial distribution in areas of Aβ deposits, suggesting a major microglial response to Aβ pathology in this mouse model. Interestingly, the Aβ42 reduction seen in mice chronically treated with both antibodies was accompanied by increased levels of sTREM2, suggesting that microglia are further activated by the ongoing therapy and involved in clearance of Aβ from the brain. In contrast, mice subjected to acute treatment and euthanized after ten weeks showed a reduction of sTREM2. This reduction was seen only in the group that showed reduced Aβ42, i.e., RmAb158-treated mice, and likely reflects a lower level of Aβ pathology [34]. This was achieved by reduction of Aβ at an earlier time point, with no direct remaining effects on microglia of ongoing treatment.

The lack of additional treatment effect of RmAb158-scFv8D3 could result from poor bioavailability due to a combination of intraperitoneal administration, rapid blood clearance, and increased blood cell interaction, leading to reduced free plasma concentration of RmAb158-scFv8D3 and hence lower brain uptake. Furthermore, repeated injection of RmAb158-scFv8D3 could alter mTfR expression in peripheral tissue or the endothelium of the BBB, leading to higher antibody uptake in peripheral organs and reduced brain uptake, respectively. It is also worth mentioning that the monospecific antibody RmAb158 did show a robust effect of chronic treatment, despite its poor brain uptake and limited distribution [19, 20]. This is in line with the clinical results obtained with its humanized version *lecanemab* [4, 6, 7] and could be a result of a longer residence time in blood, which results in a higher total exposure. In addition to a difference in blood elimination half-life, we have observed both here and in previous studies [25] that bispecific antibodies interact with blood cells, which reduces the concentration of free antibody in plasma. The effects of these processes probably contribute to a significant difference in exposure between the mono- and bispecific antibody variants and further studies will be necessary to fully understand the impact on therapeutic effect.

## Conclusions

In conclusion, this study has explored several aspects of immunotherapy with the bispecific antibody RmAb158-scFv8D3 and its monospecific variant RmAb158. Chronic administration reduced blood and brain exposure of RmAb158-scFv8D3, which in the end showed a lower therapeutic effect than expected. By studying immune responses, blood and brain exposure, and using dynamic PET imaging to visualize the biodistribution of the bispecific antibody, we could identify key mechanisms in these processes. An increased understanding of the long-term effects of treatment with bispecific, brain-penetrating antibodies will be important in future development of new antibody formats and therapeutic strategies.

## Supplementary Information

**Additional file 1: Figure S1.** Sequences of linker and scFv8D3 in RmAb158-scFv8D3 mutation variants. Amino acids in the linker between the RmAb158 IgG light chain and scFv8D3 are underlined. Mutated amino acids in linker or scFv8D3 are bold and red. **Figure S2.** ADA ELISA analysis of plasma from wt mice receiving weekly injections of RmAb158-scFv8D3 or RmAb158-scFv8D3^mut 1-3^ over the course of 7 weeks. Although not completely abolished, the ADA response against RmAb158-scFv8D3^mut 3^ appeared lower than for the other antibodies. Note the different scale in the two graphs. Each antibody was used as both capture and detection antibody for analysis of plasma from mice treated with the same antibody. **Figure S3.** Representative images of whole brain (A) and hippocampal (B) Aβ42 immunostaining of CD4+ depleted *App*^*NL-G-F*^ mice treated with PBS, low (6.4 nmol/kg) or high (32 nmol/kg) dose of RmAb158-scFv8D3, or with RmAb158 (32 nmol/kg). C. Quantification of Aβ42 immunostaining in cortex and hippocampus, expressed as integrated density (IntDen).

**Additional file 2: Video S1.** Sagittal half-body PET images obtained during 0-10 min after injection of [^124^I]RmAb158-scFv8D3^mut 3^ in wt mice. Upper panel (1-3) shows naïve mice, without pretreatment, and lower panel (4-6) shows mice immunized with the same antibody. Yellow arrow points to spleen, which was readily visible in naïve mice at the end of the scan time.

## Data Availability

The datasets generated and/or analyzed during the current study are available from the corresponding author on reasonable request.

## Abbreviations

Aβ: Amyloid-beta
AD: Alzheimer’s disease
ADA: Anti-drug antibodies
APP: Amyloid precursor protein
BBB: Blood-brain barrier
FA: Formic acid
MHC: Major histocompatibility complex
PET: Positron emission tomography
RMT: Receptor-mediated transcytosis
SPECT: Single photon emission computed tomography
scFv: Single-chain fragment variable
sTREM2: Soluble triggering receptor on myeloid cells 2
TBS: Tris-buffered saline
TfR: Transferrin receptor
