# Supplementary material for: Long-term effects of immunotherapy with a brain penetrating Aβ antibody in a mouse model of Alzheimer’s disease
Source: Alzheimers Res Ther. 2023 May 2;15:90. doi: 10.1186/s13195-023-01236-3 (PMC10152635; doi:10.1186/s13195-023-01236-3)
Supplement: Supplementary file 1 — Additional file 1: Figure S1. Sequences of linker and scFv8D3 in RmAb158-scFv8D3 mutation variants. Amino acids in the linker between the RmAb158 IgG light chain and scFv8D3 are underlined. Mutated amino acids in linker or scFv8D3 are bold and red. Figure S2. ADA ELISA analysis of plasma from wt mice receiving weekly injections of RmAb158-scFv8D3 or RmAb158-scFv8D3mut 1-3 over the course of 7 weeks. Although not completely abolished, the ADA response against RmAb158-scFv8D3mut 3 appeared lower than for the other antibodies. Note the different scale in the two graphs. Each antibody was used as both capture and detection antibody for analysis of plasma from mice treated with the same antibody. Figure S3. Representative images of whole brain (A) and hippocampal (B) Aβ42 immunostaining of CD4+ depleted AppNL-G-F mice treated with PBS, low (6.4 nmol/kg) or high (32 nmol/kg) dose of RmAb158-scFv8D3, or with RmAb158 (32 nmol/kg). C. Quantification of Aβ42 immunostaining in cortex and hippocampus, expressed as integrated density (IntDen). [file 13195_2023_1236_MOESM1_ESM.docx]

**Supplementary information, Gustavsson et al.**

**
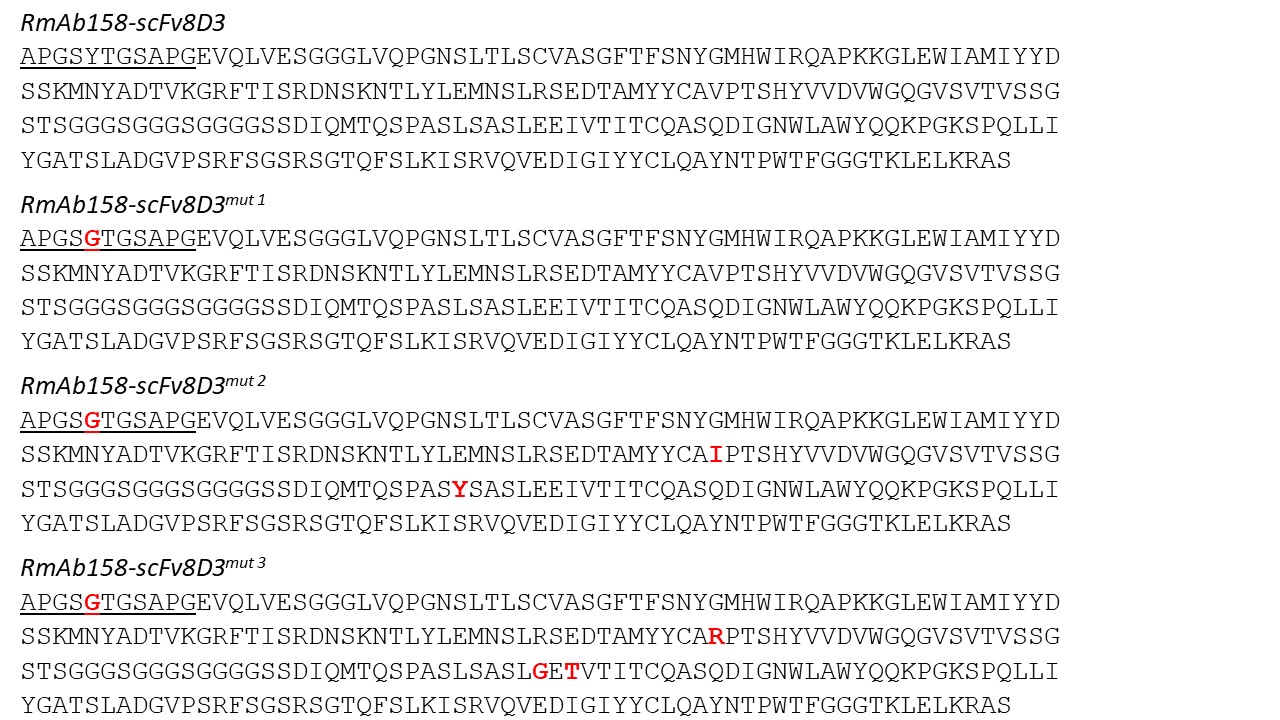
**

**Figure S1**. Sequences of linker and scFv8D3 in RmAb158-scFv8D3 mutation variants. Amino acids in the linker between the RmAb158 IgG light chain and scFv8D3 are underlined. Mutated amino acids in linker or scFv8D3 are **bold and red**.


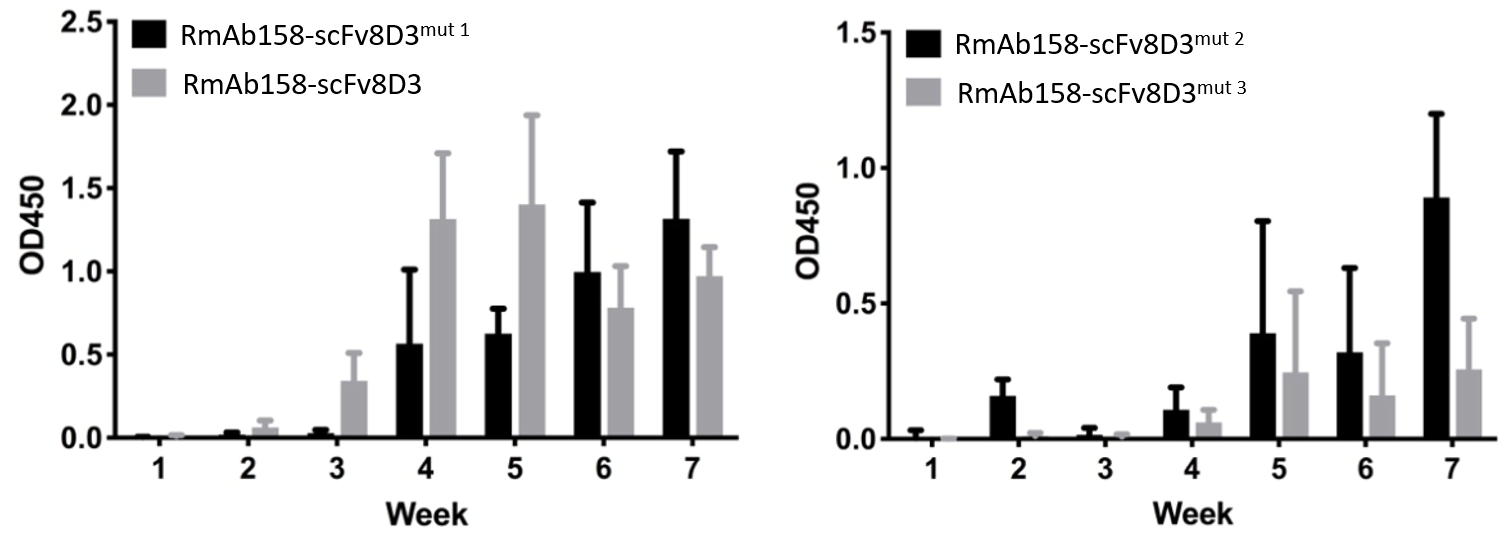


**Figure S2**. ADA ELISA analysis of plasma from wt mice receiving weekly injections of RmAb158-scFv8D3 or RmAb158-scFv8D3^mut 1-3^ over the course of 7 weeks. Although not completely abolished, the ADA response against RmAb158-scFv8D3^mut 3^ appeared lower than for the other antibodies. Note the different scale in the two graphs. Each antibody was used as both capture and detection antibody for analysis of plasma from mice treated with the same antibody.


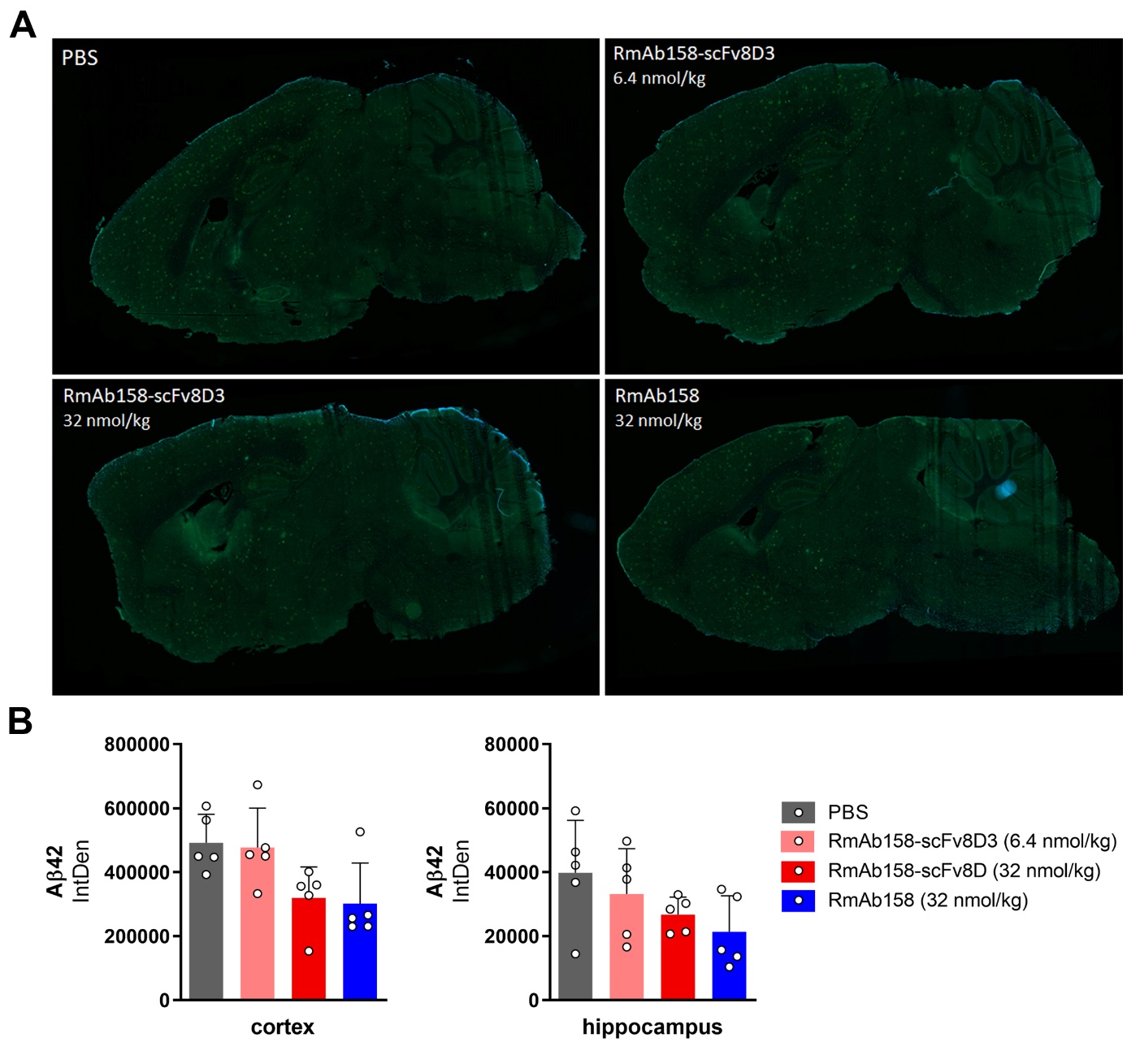


**Figure S3. A.** Representative images of whole brain Aβ42 immunostaining of CD4^+^ depleted *App^NL-G-F^* mice treated with PBS, low (6.4 nmol/kg) or high (32 nmol/kg) dose of RmAb158-scFv8D3, or with RmAb158 (32 nmol/kg). **B.** Quantification of Aβ42 immunostaining in cortex and hippocampus, expressed as integrated density (IntDen).
