# Supplementary material for: Long-term effects of immunotherapy with a brain penetrating Aβ antibody in a mouse model of Alzheimer’s disease
Source: Alzheimers Res Ther. 2023 May 2;15:90. doi: 10.1186/s13195-023-01236-3 (PMC10152635; doi:10.1186/s13195-023-01236-3)
Supplement: Supplementary file 2 — Additional file 2: Video S1. Sagittal half-body PET images obtained during 0-10 min after injection of [124I]RmAb158-scFv8D3mut 3 in wt mice. Upper panel (1-3) shows naïve mice, without pretreatment, and lower panel (4-6) shows mice immunized with the same antibody. Yellow arrow points to spleen, which was readily visible in naïve mice at the end of the scan time. [file 13195_2023_1236_MOESM2_ESM.pptx]

## Slide 1
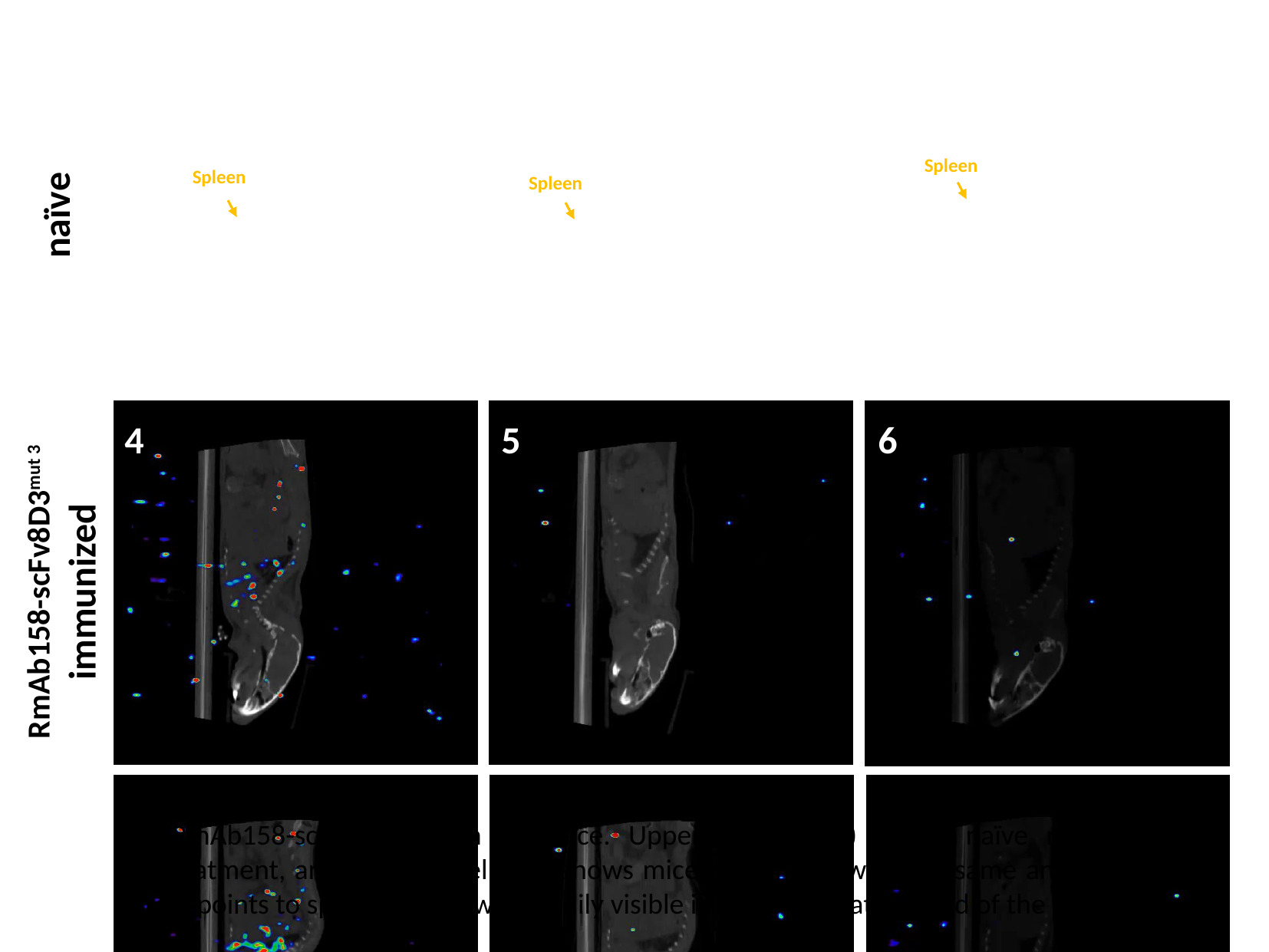

2
3
1
Spleen
Spleen
Spleen
naïve
4
5
6
RmAb158-scFv8D3mut 3 immunized
Video S1. Sagittal half-body PET images obtained during 0-10 min after injection of [124I]RmAb158-scFv8D3mut 3 in wt mice. Upper panel (1-3) shows naïve mice, without pretreatment, and lower panel (4-6) shows mice immunized with the same antibody. Yellow arrow points to spleen, which was readily visible in naïve mice at the end of the scan time.
